# The effects of onion consumption on treatment of metabolic, histologic, and inflammatory features of nonalcoholic fatty liver disease

**DOI:** 10.1186/s40200-016-0248-4

**Published:** 2016-07-22

**Authors:** Hadi Emamat, Forough Foroughi, Hassan Eini–Zinab, Mohsen Taghizadeh, Marjan Rismanchi, Azita Hekmatdoost

**Affiliations:** 1 Department of Clinical Nutrition and Dietetics, Faculty of Nutrition Sciences and Food Technology, National Nutrition and Food Technology Research Institute, Shahid Beheshti University of Medical Sciences, Tehran, Iran; 2 Department of Pathology, Taleghani Hospital, Faculty of Medicine, Shahid Beheshti University of Medical Sciences, Tehran, Iran; 3 Research Center for Biochemistry and Nutrition in Metabolic Diseases, Kashan University of Medical Sciences, Kashan, Iran

**Keywords:** Onion, Nonalcoholic fatty liver disease, Treatment, Body weight, Experimental model, Quercetin

## Abstract

**Background:**

The aim of this study was to evaluate the effects of onion powder consumption on treatment of Non-alcoholic Fatty Liver Disease (NAFLD) in an experimental model of disease.

**Methods:**

Sprague–Dawley rats were fed high-fat (HF) diet for seven weeks to induce the NAFLD. Then, they were treated by either the same diet (HF), or high-fat diet plus 7 % onion powder (HF + onion), or chow diet (control), or chow diet plus 7 % onion powder (control + onion)ad libitum for four weeks. Serum levels of fasting glucose, triglyceride, cholesterol, liver enzymes, insulin, and hepatic tumor necrosis factor-alpha (TNF-α) gene expression were determined. Hepatic histology was examined by Hematoxylin and Eosin stain.

**Results:**

Dietary food intakes and weigh gain were significantly more in animals fed control + onion diet in comparison to the other groups. Animals fed control or control + onion diet had significantly lower plasma levels of hepatic enzymes, lipid profile, glycemic indices, and hepatic TNF-α gene expression as compared with HF diet fed groups; however, there was no significant difference in the histopathologic features of NAFLD among different groups.

**Conclusion:**

Our results indicate that onion consumption can be effective in NAFLD management when it is combined with a healthy diet.

**Electronic supplementary material:**

The online version of this article (doi:10.1186/s40200-016-0248-4) contains supplementary material, which is available to authorized users.

## Background

Non-alcoholic fatty liver disease (NAFLD), as the most common liver disease, is becoming anoutstanding public health concern in the world [1]. NAFLD is strongly associated with the features of metabolic syndrome such as hyperglycemia, central obesity and dyslipidaemia [2]. It is also associated withincreased risk of all-cause mortality, contributed by liver relateddeaths as well as non-liver related causes such as malignancy,diabetes, and cardiovascular disease [1]. Although the beneficial role of diet and some dietary supplements on NAFLD treatment have been shown recently [3–9], no consensus has yet been achieved. Thus, finding effective therapies are a research priority to reduce the anticipatedburden of liver diseases [10].

Onion has the potential properties in amelioration of inflammation, hyperglycemia andhyperlipidemia [11, 12]. Its high content of flavonoids [13], makes it as a great source of antioxidants and anti-inflammatory agent [14]. It seems that all of these properties can help in treatment of NAFLD. Therefore, the purpose of thepresent study was to examine the effects of oraladministration of onion powder on hepatic and serum features of NAFLD in an experimental model of disease.

## Methods

### Animals and diets

Twenty–four male Sprague–Dawley rats (weighted 120–150 gram), which were purchased from Pasteur Institute (Karaj, Iran), were individually housed individually in wire bar-floor cages. The animals were allowed one week of acclimatization in a standard environment at 22 °C, 50 % humidity and 12-h light/dark cycles with free access to food and water. During the first week, all animals were fed a standard laboratory chow diet (Pasture Institute, Iran) and afterwards they fed a high fat, high sugar diet in seven week for induction of NAFLD [15]. Then, treatment phase was designed for four weeks. Body weights (BW) in grams were recorded on arrival and every two week thereafter. Food intakes were also monitored twice a week. In treatment phase,the animals were randomly assigned to four groups : first group fed a standard chow diet (control group) with 10 % of energy derived from fat, 30 % from protein, and 60 % from carbohydrates, second group fed a high-fat, high sugar diet (HF group) with 59 % of energy derived from fat, 30 % from carbohydrates, and 11 % from protein, the third group fed high-fat, high sugar diet added 7 %(w/w) onion powder (HF + onion group) with 59 % of energy derived from fat, 31 % from carbohydrates, and 10 % from protein and finally fourth group fed chow + onion diet (control + onion) with 10 % of energy derived from fat, 62 % from carbohydrates, and 28 % from protein. Groups were fed ad libitum. Onion powder was prepared according to the methods used by Hamauzu et al. [16]. The diets were prepared weekly and stored as vacuum packed (500 g) at −20 °C. Packs taken for use were thawed in the refrigerator at 4 °C. The food was offered daily at the beginning of the dark phase, and the remains were weighed and removed after 48 h.

After eleven-week feeding period, animals were killed in the overnight fasting state by exsanguination (under light pentobarbital anesthesia). All animal procedures were carried out in accordance with the National Nutrition and Food Technology Research Institute (NNFTRI). The study protocol was approved at NNFTRI ethics committee with ethics code of NNFTRI 1393–568.

### Tissue and blood preparation

Blood samples were collected in heparinized tubes; then, centrifuged (3500 rpm, 15 min, at 6 °C) to obtain the plasma. Fasting plasma glucose was measured immediately, and the remaining samples were kept at −80 °C before biochemical analysis.

After blood sampling, the livers were excised, washed with cold physiologic saline (0.9 %), and dried. One lobe of each liver tissue was preserved in 10 % buffered-formalin solution for histopathologic examination. Other liver samples (200 mg) were placed in liquid nitrogen tank, and then kept at −80 °C for gene expression evaluation [17–20].

### RNA extraction and quantitative RT-PCR

Total RNA was purified using RNeasyPlus Mini Kits (Qiagen) according to the manufacturer’s instructions and cDNA synthesized with Superscript II reverse transcriptase (Invitrogen). Quantitative real-time PCR was performed using the Bio-Rad Laboratories MJ mini Opticon Real-Time PCR System, using IQ SYBR Green Supermix (Bio-Rad).

The PCR mix contained 2 μl cDNA, 1 μl of the appropriate forward and reverse primers, and 2 μl SYBR Green PCR Master mix in a total volume of 25 mL. PCR consisted of 50 cycles of denaturation at 94 °C for 30 s, annealing at melting temperature (Tm) for 30 s, and extension at 72 °C for 60 s. Primer sequences for each target gene, their source as well as their optimal PCR annealing temperatures are as follows: GAPDH forward primer 5′-GTGCTGAGTATGTCGTGGAGTCTA-3′ and reverse 5′- TCTCGTGGTTCACACCCATCAC −3′ (Tm 60 °C), and TNF-α forward primer 5′- ACT GAA CTT CGG GGT GAT TG −3′ and reverse 5′- GCT TGG TGG TTT GCT ACG AC −3′ (Tm 60 °C). Primer specificity was confirmed from the product size by agarose gel electrophoresis and the specificity of the PCR products checked by melt curve analysis.

### Biochemical assessments

Plasma concentrations of alanine aminotransferase (ALT), and aspartate aminotransferase (AST) were measured using optimized UV at 340 nm, and insulin concentrations were measured using a rat insulin radioimmunoassay kit at 4 °C (Linco Research Inc, St Charles, MO). Plasma glucose and triglyceride were measured colorimetrically, Gamma glutamyltransferase (GGT), and Alkaline phosphatase (ALP) assessed photometrically, total Cholestrol, HDL, and LDL cholesterol were examined enzymatically all by using a commercial kit (Parsazmoon, Tehran, Iran).

### Histopathology

Five sections from different lobes of each liver were submitted and processed through ethyl alcohol and xylene series, and embedded in paraffine blocks. Slides were stained with Hematoxylin and Eosin and Masson's Trichome and viewed under light microscopy by Nikon E 200. The grading was defined as follow: for hepatic steatosis: grade 0, no fat; grade 1, steatosis occupying less than 33 % of the hepatic parenchyma; grade 2, 34–66 % of the hepatic parenchyma; grade 3, more than 66 % of the hepatic parenchyma; for inflammatory cell infiltration: grade 0: none; grade 1, 1–2 foci/field; grade 2, 3–4 foci/field; grade 3, more than 4 foci/field [20]; for ballooning: minimal, mild, and marked [21].

### Statistical analysis

Results are expressed as median (interquartile range) and using nonparametric tests included Mann Whitney, Kruskal Wallis and chi-square tests. *P* < 0.05 was considered for significance level. All statistical analyses were performed with the use of either GraphPad Prism Software Version 5.00 (GraphPad Software, SanDiego, CA), or SPSS 20.0 software (Chicago, IL, USA). The raw data are shown in Additional file 1.

## Results

**Fig. 1:**
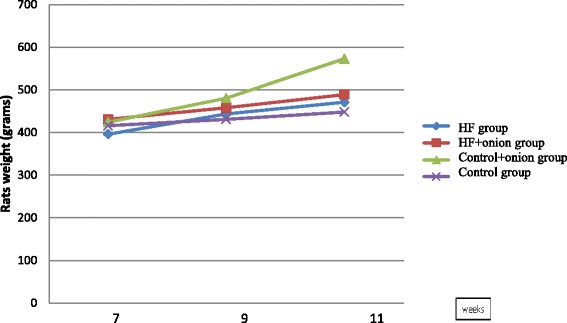
Animal weights in different groups during study. Weight gain was statistically different between four groups (*P <* 0.05). Weight gain was significantly more in control + onion group than the other three groups (*P <* 0.05). HF group weight gain was more than control group (*P* = 0.002). Other differences were not statistically significant between groups

Weight gain was measured and compared between four groups in beginning, second and fourth weeks of treatment phase (Fig. 1). Weight gain was statistically different between four groups (*P <* 0.05). Weight gain was significantly more in control + onion group than the other three groups (*P <* 0.05). Also, HF group weight gain was more than control group (*P* = 0.002). Other differences were not statistically significant between groups.

**Fig. 2:**
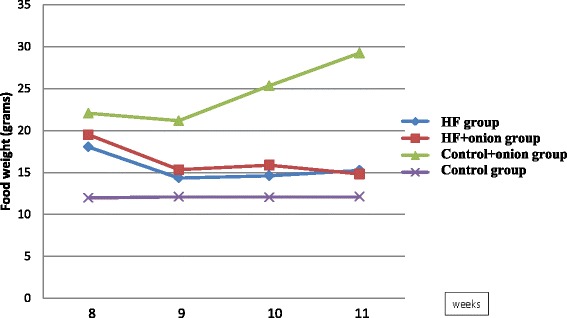
Food intakes in different groups during the study. Food intake was significantly different between four groups in every week (*P <* 0.01). Food intake was significantly more in control + onion group than other three groups (*P <* 0.01). Food intake in control group was less than food intake in HF group and HF + onion groups (*P <* 0.01). No difference was seen between HF and HF + onion group in food intake

Figure 2 shows the food intakes of different groups during the study. Kruskal–Wallis test showed that food intake was significantly different among four groups in every week (*P <* 0.01). Food intake was significantly more in control + onion group than other three groups (*P <* 0.01). Food intake in control group was less than food intake in model group and HF + onion groups (*P <* 0.01).No difference was seen between HF and HF + onion group in food intake.

Table 1 shows the plasma levels of hepatic enzymes, glycemic, and lipid profile in the four groups at the end of the study. HF group had significantly higher plasma level of AST (*P* = 0.05),TG (*P* = 0.01), glucose (*P* = 0.02), cholesterol (*P* = 0.04) and ALP (*P* = 0.004) compared with control group. Adding onion to control group decreased ALT in comparison with HF group(*P* = 0.02). Control and control + onion groups were similar except in TG that was less in control + onion compared to control group (*P* = 0.02). Adding onion to HF diet could not cause any significant changes as compared with HF diet.

**Table 1:** Serum level of hepatic enzymes, lipid and glycemic profiles in different groups at the end of study

	HF group (median (IQR))	HF + onion group (median (IQR))	Control group median (IQR))	Control + onion group (median (IQR))	*P*-value^d^
ALT(IU/L)	57 (47.2–75.7) ^b,c^	62.5 (47.5–76.7) ^c^	50 (29.2–62.7) ^a,b,c^	39 (30.7–49) ^a^	0.05
AST(IU/L)	34 (28–50) ^b^	25 (19.5–35) ^a,b,c^	21.5 (18.5–33) ^a,c^	23.5 (18.7–31.2) ^c^	0.1
GGT (IU/L)	3.9 (3.4–4.6) ^a^	2.9(2.2–3.8) ^a^	3 (2.7–3.2) ^a^	2.8 (2.4–3.4) ^a^	0.1
ALP (IU/L)	890 (754.7–1002)^a^	937 (812–1098)^a^	468 (411–542) ^b^	479 (395–635) ^b^	0.002
Glucose (mg/dl)	194 (160–215.7) ^b^	163 (134.7–189.5) ^a,b,c^	150.5(135.2–168.5) ^a,c^	133 (118.5–151) ^c^	0.04
Insulin (pmol/l)	465 (402–512)^a^	444 (392–506)^a^	192 (176–219)^b^	179 (165–202)^b^	0.01
TG (mg/dl)	135 (118–145) ^a^	124.5 (114.2–149) ^a,b^	102 (88.5–121.5) ^b,c^	83 (70.2–93.2) ^c^	0.001
Cholesterol (mg/dl)	125 (110.7–138)^a^	122.5 (116–139.7)^a^	109.5 (98–116) ^b^	104 (96–118.5) ^b^	0.03
HDL-C (mg/dl)	45.5 (31.5–53.5) ^a^	37.5 (29.2–43) ^a^	41.5 (36.5–52.2) ^a^	50.5 (43.7–56) ^a^	0.1
LDL-C (mg/dl)	56.7 (36.7–75.6) ^a,b^	62.5 (51.2–80.4) ^b^	44.9 (33.3–52.5) ^a,b^	39.8 (33.6–50.3) ^a^	0.08

*IQR* interquartile range, *ALT* alanine aminotransferase, *AST* aspartate aminotransferase, *GGT* gamma glutamine transferase, *ALP* alkaline phosphatase, *TG* triglyceride, *HDL-C* high density lipoprotein cholesterol, *LDL-C* low density lipoprotein cholesterol

^a,b,c^In every row different scripts show significant difference

^d^Kruskal–Wallis test

**Fig. 3:**
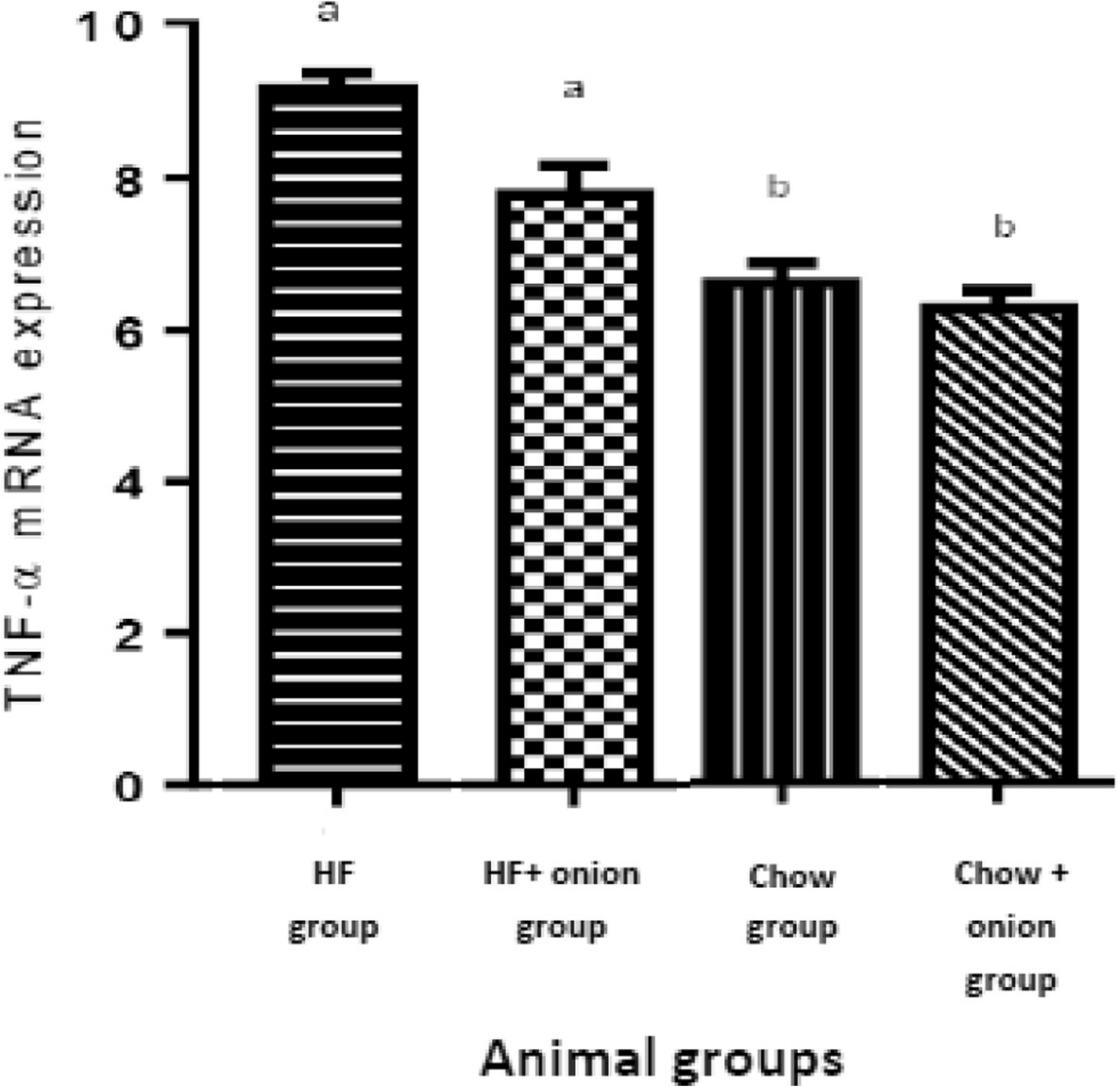
Hepatic TNF-α m RNA expression comparison among four groups. Different scripts show significant difference

Hepatic TNF-α gene expression was not significantly decreased in HF + onion group in comparison to HF group. Treatment by chow or chow + onion could significantly reduce hepatic TNF-α gene expression (P < 0.05) (Fig. 3).

**Fig. 4:**
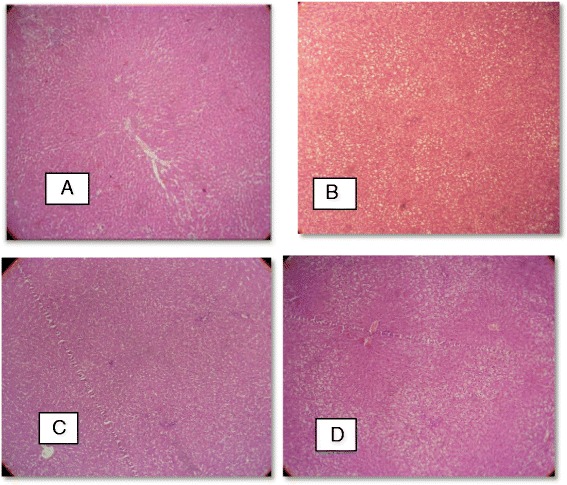
Hepatic pathology in rats fed control + 7 % onion × 100 (**a**) fed high fat, high sugar diets ad libitum × 100 (**b**) fed chow diet ad libitum × 100 (**c**) and fed high fat, high sugar diets + 7 % onion × 100 (**d**). The liver samples were stained with Hematoxylin and Eosin

As shown in Table 2 and Fig. 4, no significant difference was seen among four groups in hepatologic indices (steatosis, ballooning, lobular inflammation and portal inflammation) after four weeks of treatment.

**Table 2:** Histopathological characteristics of HF, control, HF + onion and control + onion groups (*n* = 6 in each group)

	Group	Stage		*P* value
		Low	High	
Steatosis	Control	6 (100)	0	0.06
n (%)	HF	3 (50)	3 (50)	
	HF + onion	5 (83.3)	1 (16.7)	
	Control + onion	6 (100)	0	
Ballooning	Control	3 (50)	3 (50)	0.3
n (%)	HF	1 (16.7)	5 (83.3)	
	HF + onion	4 (66.7)	2 (33.3)	
	Control + onion	3 (50)	3 (50)	
Lobular inflammation	Control	5 (83.3)	1 (16.7)	0. 5
n (%)	HF	3 (50)	3 (50)	
	HF + onion	4 (66.7)	2 (33.3)	
	Control + onion	5 (83.3)	1 (16.7)	
Portal inflammation	Control	4 (66.7)	2 (33.3)	0. 5
n (%)	HF	3 (50)	3 (50)	
	HF + onion	3 (50)	3 (50)	
	Control + onion	5 (83.3)	1 (16.7)	

Low = 0 or 1 stage

High = 2 or 3 stage

*HF* high fat

## Discussion

Our results have shown that onion consumption can attenuate the risk factors of NAFLD; however, these effects were not statistically significant without accompanying with a healthy diet. Onion can ameliorate the lipid profile, glycemic, and inflammatory indices through its antioxidant, anti-inflammatory, hypoglycemic and hypolipidemic effects [16, 22–26], but these improvements were not statistically different from control group because the animals who consumed onion, their food intake and weight gain were more than those without onion supplementation. Since body weight plays a pivotal role in the pathogenesis of NAFLD [5], it is possible that those animals who received onion, liked its taste and ate more foods, resulting to more weight gain so that the beneficial effects of onion were weakened due to the adverse effects of high food consumption and weight gain.

The animals in control groups whom fed the chow diet with or without onion had significantly lower plasma levels of hepatic enzymes, glycemic and lipid indices, and hepatic TNF-α gene expression, which confirms the pivotal role of diet and weight in the NAFLD management [5]. The effects of onion on the growth performance and appetite enhancement have been shown previously [27, 28]; however, some of its components act inversely and reduce the body weight [29]. Our results confirm that the whole onion can be used as a growth promoter; however, some of its components may have anti obesity properties.

## Conclusion

In conclusion, our results indicate that adding onion to the diet increases the dietary intakes and weight gain, which contradicts its antioxidant, anti-inflammatory, hypoglycemic and hypolipidemic effects in NAFLD management. Thus, onion consumption can help in NAFLD management when it is combined with a healthy diet. Further studies are needed to explorethe effects of different types of onion on NAFLD management.

## Abbreviations

ALP, alkaline phosphatase; ALT, alanine aminotransferase; AST, aspartate aminotransferase; GGT, gamma glutamine transferase; HDL-C, high density lipoprotein cholesterol; HF diet, high fat diet; IQR, interquartile range; LDL-C, low density lipoprotein cholesterol; NAFLD, non-alcoholic fatty liver disease; TG, triglyceride

## Additional file

Additional file 1: The raw data. (XLS 96 kb)
